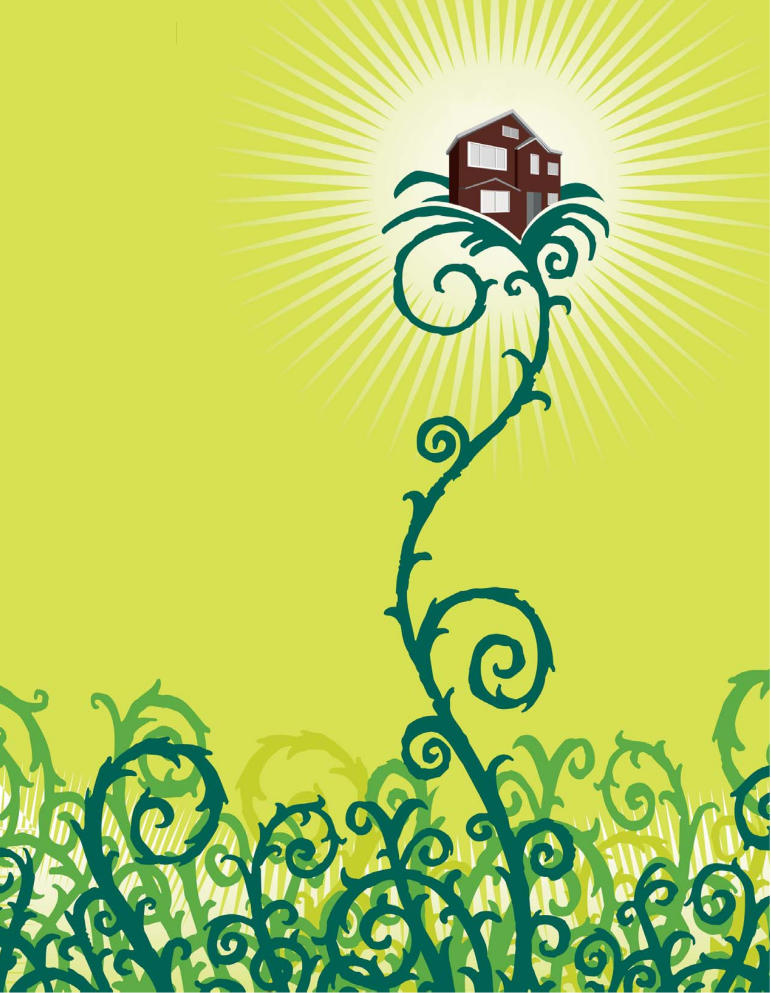# Room to Grow: Incentives Boost Energy-Efficient Homebuilding

**DOI:** 10.1289/ehp.116-a32

**Published:** 2008-01

**Authors:** Charles W. Schmidt

“Home is where the hearth is,” goes an old play on words. But what about the solar panel or the wind turbine? Maybe they could be where the home is, too. That’s the view of a growing movement to make energy-efficient homes the norm, not the exception. Such homes currently constitute just a tiny fraction of the U.S. residential market. Now, driven by mounting fuel costs, heightened sensitivities to climate change, and rising worries over indoor air quality, the race is on to reform housing for better environmental performance.

Decades ago, U.S. state and local governments began offering limited incentives to motivate energy-efficient homebuilding, and most programs today are still limited to energy conservation; consumers looking for breaks on other green features—for instance, non-toxic or recycled building materials, or rain collection barrels for lawn and garden watering—are largely out of luck, at least for now. Only New Mexico offers a comprehensive incentive package that goes beyond energy to address other green concerns. A few other states are broadening their programs, but slowly, says Jason Hartke, the manager for state and local advocacy for the nonprofit U.S. Green Building Council (USGBC).

As far as energy efficiency incentives go, the number and variety on offer has swelled dramatically in recent years. In places like Aspen, Chicago, Tucson, Tempe, Santa Monica, Scottsdale, Seattle, and Washington, DC, among others, building departments provide leadership in green residential design, says Darren Meyers, contracts manager for the International Code Council (ICC), a membership association based in Washington, DC. Many of these cities have adopted policies that spur architects, homebuilders, and code enforcers to incorporate green building practices.

Most incentive programs today are limited to energy conservation. Only New Mexico offers a comprehensive package that goes beyond energy to address other green concerns. A few other states are slowly broadening their programs.

Today, the federal government and a growing number of utilities and private organizations offer incentives with real value. In some states, their combined worth can offset the cost of a solar power installation by more than 50%. Tax credits, utility rebates, expedited permitting for new construction, even coupons for energy-efficient products (compact fluorescent lamps, for example) are jump-starting the market, ideally in a sustainable way. “These incentives are an important step toward making [energy efficiency] standard in residential settings,” says Edward Legge, a spokesperson for the Edison Electrical Institute, a utility trade association in Washington, DC.

## Jump-Starting the Market

Such incentives have their roots in the energy efficiency movement of the 1970s. Stunned by Middle East oil shocks, Congress devised efficiency standards that eventually morphed into the International Energy Conservation Code (IECC) and American Society of Heating, Refrigerating, and Air-Conditioning Engineers (ASHRAE) Standard 90.1. Both codes are fundamental cornerstones for energy efficiency in building design, insulation, heating, cooling, hot water, and lighting.

Both the IECC and ASHRAE 90.1 are viewed as minimum standards; to qualify for incentives, products and homes have to exceed them. For instance, to qualify for the Energy Star label, products must exceed the IECC standard by at least 30%. Energy Star was created by the Energy Policy Act in 1992. Today, it’s co-administered by the U.S. Environmental Protection Agency (EPA) and the Department of Energy (DOE). Over time, Energy Star’s purview has grown to include appliances, office equipment, lighting, electronics, and even single-family homes. But until recently, consumers who bought Energy Star products had only their conscience to motivate them—there weren’t any financial incentives behind the program, and its growth overall was slow.

That changed in 2005, when revisions to the Energy Policy Act tied federal tax credits to purchases that exceeded Energy Star criteria. These credits became the first—and remain among the only—federal incentives for energy-efficient products and homes. Consumers can now claim federal income tax credits ranging from a low of $150 for an Energy Star furnace or boiler to $2,000 for an Energy Star–rated house. Richard Faesy, a senior project manager with the nonprofit Vermont Energy Investment Corporation, says these incentives have boosted Energy Star sales, thus affirming their market value.

The credit for an Energy Star–rated house goes directly to the builder (or to owners who manage the construction of their own home), depending on inspection results by a “home energy rater” who is, in turn, certified by the Residential Energy Services Network (RESNET), a coalition of energy efficiency home inspectors headquartered in Oceanside, California. Meyers says home energy raters use what’s known as a “blower door test” to evaluate living spaces for drafts and air leaks. “Basically, they close windows and doors and bring the house to a known standard pressure,” he explains. “That allows the rater to test for air leaks, which translate to a measure of how tight the overall construction is. . . . Home energy raters also evaluate windows, doors, ducts, insulation placement, and mechanical equipment to come up with an overall score for the home.” Homes qualify for the $2,000 Energy Star credit if they exceed the IECC benchmark rating by 50% or more (or 30% or more for manufactured homes).

In the grand scheme of things, Energy Star credits might seem rather small. But they actually cover a surprising portion of what it costs to build green. The expense of a tight building envelope, better windows, and energy-efficient systems generally adds just $3–5 per square foot and often less, according to Greg Kats, managing director of Good Energies, a venture capital firm that invests in sustainable technology. Assuming the lower end of that range, Energy Star credits combined could conceivably cover half the “green premium” of a sustainably built 2,000-square-foot home. [For more on this topic, see “Bringing Green Homes Within Reach: Sustainability Meets Affordability,” p. A24 this issue.]

A hidden cost in that equation, however, is that of the Energy Star inspection itself, which can run from a few hundred to a few thousand dollars. Fortunately, programs in a growing number of states, including most of New England, Texas, and California, will often pay that cost. Consumers who want to investigate their options can access the Database of State Incentives for Renewables and Efficiency, a comprehensive online catalog of organizations that help subsidize sustainable building.

## Beyond the Feds

Utilities are a growing and often overlooked source of financial incentives for energy-efficient homes. “Across the board, the utility industry is working to grow its incentives programs,” Legge says. “It makes business sense for us to recover costs and to make a return from effective efficiency efforts.” The most progressive utilities, he adds, help pay for compact fluorescent lamps, more efficient boilers and furnaces, even appliances. The Long Island Power Authority (LIPA), a New York–based utility, actually buys Energy Star refrigerators for residents who make less than 70% of the local median income.

LIPA and other forward-thinking utilities also contribute toward residential purchases of solar power. A full solar installation of up to 5 kilowatts might cost more than $30,000 (of course, homeowners can buy less solar capacity and combine it with grid power). But LIPA’s program covers nearly half that amount, regardless of the homeowner’s income. What’s more, the state of New York kicks in a 25% tax credit capped at $5,000, to which the federal government—through Energy Star—adds a 30% tax credit capped at $2,000. All this is in addition to the previously discussed Energy Star incentive for achieving a high-efficiency home energy rating. Thus, the cost for a full solar installation might drop to slightly more than 30% of its retail value.

At the state level, New Mexico is unique in that it rewards green home-building comprehensively. Stace McGee, president of Environmental Dynamics, a sustainable architecture firm in Albuquerque, says other states including Nevada, Florida, Oregon, and New York have considered broader incentives that—like New Mexico’s—go beyond energy, but they haven’t yet taken the plunge.

To be sure, New Mexico is starting small. The entire package, passed in March 2007, is capped at $5 million per year each for residential and commercial construction. McGee emphasizes the cap was necessary to reassure state legislators that green incentives wouldn’t be paid with an open checkbook. “It was an easy way for everyone to buy into the program,” he explains. “Over time, we might be able to increase the amount, but for now, this was something everyone could agree on.”

The amounts paid under New Mexico’s program rise as homes incorporate more green design features. For instance, a silver-certified home under the USGBC Leadership in Energy and Environmental Design (LEED) rating system qualifies for tax credits of $5.00 per square foot for the first 2,000 square feet, while a top-tier LEED platinum-certified home qualifies for $9.00 per square foot for the first 2,000 square feet; the credit is halved for additional floor space. McGee predicts the average homeowner can bank as much as $10,000 in credits, a substantial sum that could go a long way toward funding green designs.

## Barriers to Energy-Efficient Building

But even as states and utilities promote energy-efficient homebuilding, other barriers remain. In some cases—particularly in historic districts—homeowners face zoning restrictions on green buildings that don’t match the local character. In Portland, Maine, for instance, one family spent 8 months battling zoning officials before gaining permission to build a south-facing structure built with recycled fiber cement siding. Unlike the surrounding homes in this waterfront working-class neighborhood, the new house resembles a massive, square block outfitted with enormous triple-glazed windows.

Until recently, consumers who bought Energy Star products had only their conscience to motivate them. That changed in 2005, when revisions to the Energy Policy Act tied federal tax credits to purchases that exceeded Energy Star criteria.

But energy-efficient homes needn’t look unusual to run into zoning problems. All across the country, local homeowners’ associations (HOAs) have blocked residents from installing solar panels, insulated windows, even clotheslines—perhaps the most inexpensive of energy-saving additions—in order to maintain neighborhood standards for appearance. According to the 18 September 2007 *Wall Street Journal*, only 10 states currently limit HOAs’ ability to restrict the installation of solar power systems or assign that power to local authorities (and except for Florida, Hawaii, and Utah, it is unclear in these states whether clotheslines qualify as “solar devices”).

Meanwhile, high-density “smart growth” developments that link sustainable housing with public services face perpetual zoning problems, says Ed McMahon, a senior resident fellow at the Urban Land Institute, a nonprofit research and outreach organization in Washington, DC. “No one’s looking at the big picture: mixed-use projects that unite housing and services are inherently green because people drive less,” he explains. “Most zoning regulations favor conventional single-use, suburban development.” Along those lines, it took years for EcoVillage, a cohousing development in Loudoun County, Virginia, to circumvent zoning restrictions that require much larger lot sizes than those proposed for the “cluster” dwellings in this community, according to McMahon.

A number of ongoing initiatives now seek to address those concerns. The Community Associations Institute, an Alexandria, Virginia–based organization that represents HOAs, recently began an initiative to promote green designs for development communities and condominiums, says spokesperson Frank Rathbun. There are currently 300,000 HOAs nationwide, representing a combined population of nearly 60 million people. “Our goal will be to help community associations across the country develop approaches to sustainability,” Rathbun says. “Each community has to decide what’s appropriate based on its own merits.”

Manufacturers are also getting into the game, doing their part to normalize energy efficiency. Atlantis Energy Systems of Poughkeepsie, New York, for instance, has broadened a line of solar panels that resemble roof tiles, making them less obtrusive than standard panel arrays.

The combined influence of these efforts—linked to market forces that make energy-efficient homes environmentally, economically, and aesthetically attractive—have a long way to go to transform traditional housing. But the seeds of change have been planted, and a nascent transformation appears to be under way.

## Figures and Tables

**Figure:**